# Dr. C. S. Osborne

**Published:** 1913-01

**Authors:** 


It is earnestly requested that friends of deceased physicians in western New York or of those having affiliations in this region, send us brief obituary notices or notify the family of the deceased of our willingness to publish such notices. Such notices should be sent as promptly as possible and may be accompanied by photographic reproductions.

---

Dr. C. S. Osborne of Ozone Park, Borrough of Queens, died Dec. 11 of cerebral apoplexy. He was a resident of Leroy in early life.

